# Development and application of the condom catheter method for non-invasive measurement of bladder pressure

**DOI:** 10.4103/0970-1591.45546

**Published:** 2009

**Authors:** R. van Mastrigt, J. J. M. Pel, J. W. N. C. Huang Foen Chung, S. de Zeeuw

**Affiliations:** Sector Furore, Department of Urology, Erasmus MC, The Netherlands

**Keywords:** Urodynamics

## Abstract

**Objectives:**

A non-invasive method to measure the bladder pressure in males using a condom catheter has been developed. The measurement technique, its validation and limitations, a diagnostic nomogram to non-invasively diagnose bladder outlet obstruction (BOO), and results of large-scale application are discussed.

**Methods:**

Modified incontinence condoms are attached to the penis. During voiding the flow of urine is mechanically interrupted. The subsequent maximum pressure in the condom reflects the isovolumetric bladder pressure. The method was validated in a group of 46 patients with lower urinary tract symptoms who were simultaneously studied invasively and non-invasively. Subsequently it was applied in a non-invasive epidemiological study in 1020 healthy males.

**Results:**

The reproducibility of the measured isovolumetric bladder pressure is comparable to that of conventional pressure-flow parameters. The measured pressure can be used to diagnose bladder outlet obstruction with a diagnostic accuracy (Area Under receiver operator characteristic curve) of 0.98, which compares most favorably with the area under the curve of 0.79 of Q_max_ in the same population. During condom catheter measurements, both the involuntary interruption of voiding and the forced diuresis increase post-void residual volume. This increase does not affect the accuracy of the pressure measurements.

**Conclusions:**

We conclude that in males bladder pressure can successfully be measured non-invasively using the condom catheter method. By combining the measured volumetric bladder pressure with a separately measured free flow rate, BOO can non-invasively and accurately be diagnosed.

## INTRODUCTION

In most ageing males, the prostate enlarges which may obstruct the urethra that it surrounds. As a result, the flow rate may be reduced, voiding becomes more frequent and the risk of residual urine in the bladder after voiding increases. A weakly contracting detrusor, however, may also reduce the flow rate. To differentiate between both causes of impaired voiding, the bladder pressure needs to be measured. The International Continence Society (ICS) has recommended a provisional method for diagnosing obstruction on the basis of bladder pressure measured via catheters in the bladder and rectum.[1] The invasiveness of these measurements, however, limits the clinical application of this test and scientific research. Starting at the end of the previous century, non-invasive methods to assess bladder function have been developed.[2] The sector Furore of the Erasmus MC developed an external condom catheter to measure the bladder pressure non-invasively. This article presents an overview of the development and application of this condom-type catheter.

## A SET UP FOR LARGE-SCALE APPLICATION OF THE CONDOM CATHETER METHOD FOR NON-INVASIVE URODYNAMICS

Several prototypes of the catheter have been developed and tested in the past.[3–5]

**Figure 1:**
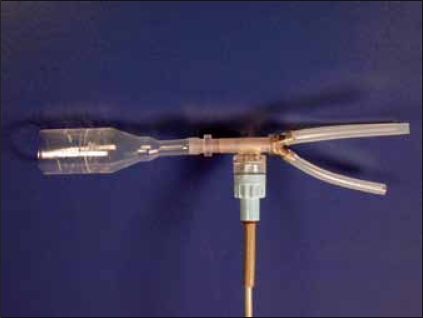
The main parts of the condom catheter measurement system: a modified incontinence condom, a dome with three metal outflow conduits, and a reusable pressure transducer. Only two of the three outflow conduits are visible.

**Figure 2:**
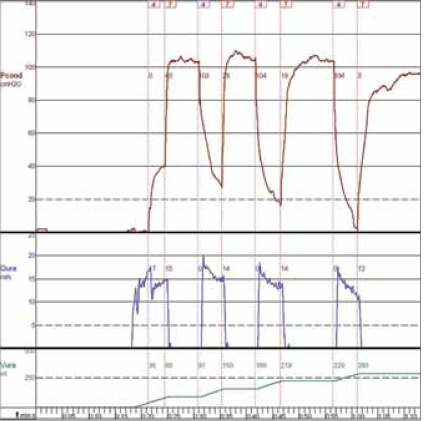
An example of a condom catheter measurement: The upper trace shows the pressure measured in the condom, the middle trace the urinary flow rate, and the bottom trace the voided volume. The numbered flags indicate the position of the valves in a binary code. 0 means all valves are open, outflow resistance is minimal. 7 means all valves are closed, effectively interrupting the urinary flow. Intermediate values offer intermediate resistance to flow.

Figure 1 shows the key elements of the latest version, intended for large-scale application. Modified incontinence condoms (Rochester Medical Corp®) are connected to a modified disposable pressure dome attached to a reusable Statham® pressure transducer. The dome has three metal outflow conduits, with different caliber. Tubing is attached to the outflow conduits, and is led through three pneumatic valves that enable closure of the tubes by compression. The tubes drain into a Dantec® urinary flow meter. By opening and closing the valves in different combinations, 8 different levels of outflow resistance can be applied; including a condition in which all valves are closed and flow through the system is effectively interrupted. The valves are controlled by a PC with software developed in Labview®, which also records the urinary flow rate and pressure. Figure 2 shows an example of a measurement done with this setup. After the onset of voiding, the outflow resistance was increased, by closing some valves. As a result, the condom pressure rose to approximately 40 cmH_2_O. Subsequently all valves were closed and pressure rose to an equilibrium of 105 cmH_2_O. At this point, there was no flow through the urethra so that this pressure represents the isovolumetric bladder pressure. Subsequently some valves were opened and flow re-established. The interruption was repeated several times, and the highest measured pressure (excluding artefacts) was considered a measure for the urinary bladder contractility.

### Validation of the measurement technique

The method was first tested in a group of healthy volunteers.[6] All were able to apply the condom and the laboratory film correctly. None of them showed inhibition of the flow rate after a single interruption of the stream so that more than one pressure reading could be taken in one voiding.

**Figure 3:**
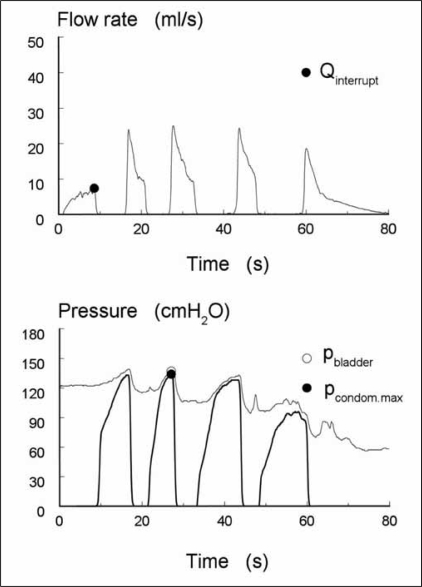
The flow rate (top panel) and the simultaneously measured bladder pressure (thin line; transurethral catheter) and condom pressure (thick line) in an obstructed patient (lowest panel). The maximum bladder pressure and condom pressure are high and correlate well. The interrupt flow rate is about 8 ml/s.

In a group of 43 patients with lower urinary tract symptoms (LUTS), we tested how well the condom pressure reflected the bladder pressure.[4] First, all patients underwent a standard pressure-flow study (PFS). On the basis of this test with catheters in the bladder and rectum, the patients were classified as non-obstructed, equivocal or obstructed using the provisional ICS method for definition of obstruction.[1] Then, in a second procedure, the condom catheter was adjusted to the penis leaving the transurethral catheter in situ. After filling the bladder, the transurethral catheter was connected to a pressure transducer to measure the bladder pressure simultaneously with the condom pressure. Figure 3 shows an example, with the interrupted flow rate in the top panel and the simultaneously measured pressures in the lowest panel. The interrupt flow rate was about 8 ml/s. During the second interruption, the maximum pressures were measured and both correlated well (∼135 cmH_2_O). The median difference between the pressure in the bladder and that in the condom was 11 cm H_2_O, which was partly caused by a height difference between both transducers.

A previous study showed that on the basis of a combination of isovolumetric bladder pressure and maximum flow rate, classification of bladder outlet obstruction (BOO) is possible.[7] Using the invasive pressure-flow test, 46 patients were therefore stratified in two groups: (1) a combined non-obstructed and equivocal group and (2) an obstructed group.

**Figure 4:**
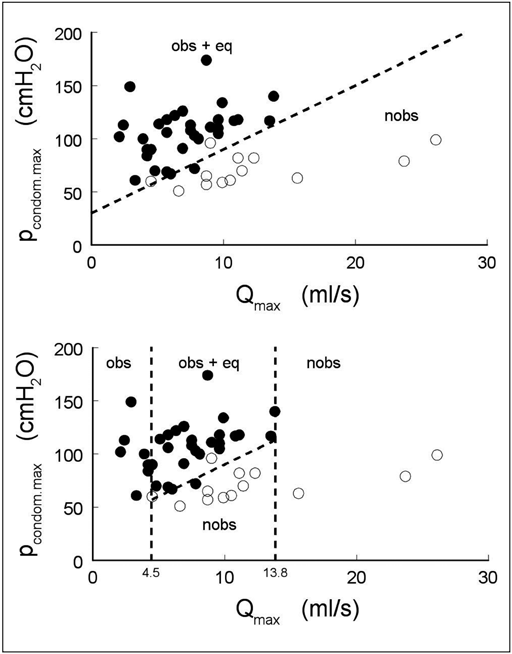
Top panel, the maximum condom pressure is plotted vs. the maximum flow rate and the combined obstructed and equivocal patients (closed circles) are separated from non-obstructed patients (open circles). Bottom panel, a two-step approach: patients with a maximum flow rate less than 4.5 ml/s or higher than 13.8 ml/s, are classified by flow rate alone. The remaining patients are classified using a combination of maximum flow rate and a separately measured maximum condom pressure.

**Figure 5:**
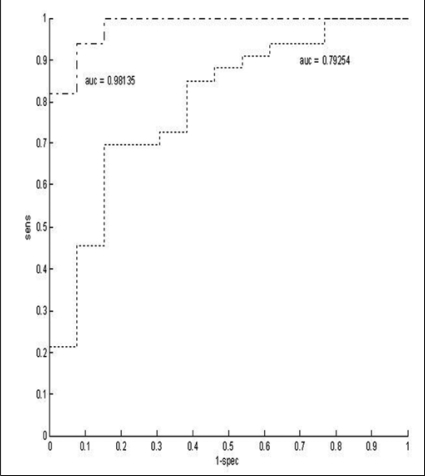
Receiver Operating Characteristic (ROC) curve of the non-invasive condom pressure measurement (dot-dashed line, with area under the curve (AUC)=0.98). The sensitivity of the test in discriminating obstructed patients from non-obstructed and equivocal patients was plotted as a function of 1-specificity. The AUC reflects the percentage of randomly drawn pairs of patients (one from the obstructed group, one from the not obstructed group) that is correctly diagnosed by the test, and is a measure for the accuracy of the test. The dotted line (with AUC=0.79) is the ROC curve for using the maximum flow rate Qmax for diagnosing obstruction in the same group of patients.

We constructed a nomogram based on flow rate and condom pressure[8] using logistic regression to calculate a separation line, and found that 91%, i.e., 42 of the 46 patients could be classified correctly, see Figure 4, top panel. By parallel shifting of the separation line up- and down-wards we calculated the ROC (Receiver Operating Characteristic) shown in Figure 5. The area under the curve (AUC) reflects the percentage of randomly drawn pairs of patients that is correctly diagnosed by the test, and is a measure for the accuracy of the test. The AUC of 0.98 signifies an excellent test. It should be noted that this AUC was calculated from a limited population of 46 patients and that the separation line shown in Figure 4 was calculated from and applied to the same group. Both factors tend to increase the value found for the accuracy of the test. This value also depends on the properties of the population, i.e., on the distribution of the patients on the nomogram. Therefore, we included a second ROC curve in Figure 5, showing the accuracy of using only the maximum flow rate Q_max_ as a criterion for diagnosing the patients. Earlier we have shown that when patients are evenly distributed on pressure flow nomograms, the AUC of Q_max_ as a test for obstruction equals 0.619.[9] In Figure 5 the AUC of Q_max_ is 0.79, implying that the distribution of the population of patients used positively biases the AUC. However, the AUC of the non-invasive condom test is much higher than that of Q_max_ confirming that this test is much better for diagnosing obstruction than Q_max_.

The patient population used had a wide variety of urological symptoms ranging from BOO to incontinence. We found that in this population, all patients voiding with a maximum flow rate smaller than 4.5 ml/s were obstructed and all those voiding with a maximum flow rate higher than 13.8 ml/s were non-obstructed. Therefore, a two-step approach could be efficient: Patients voiding with a Q_max_ < 4.5 ml/s and Q_max_ >13.8 ml/s are classified by flow rate alone. The remaining patients are classified using a combination of maximum flow rate and a separately measured maximum condom pressure, see Figure 4, lowest panel. Using this strategy, 30% of the patients could correctly be classified on the basis of Q_max_ alone. To diagnose BOO in the remaining 70% of the patients, non-invasive measurement of bladder pressure is necessary.

## LIMITATIONS OF THE MEASUREMENT TECHNIQUE

Patients and volunteers were asked not to strain during voiding. Despite this encouragement, some patients did. In a small group, we observed that in some cases the relatively high abdominal pressure was not reflected in the pressure measurement in the condom. Obviously, in these cases straining led to closure of the urethra resulting in an unreliable pressure reading in the condom. We therefore concluded that this test could only be done in those who void without straining. Furthermore, a too low flow rate at the moment of interruption prolongs the filling of the condom, which increases the risk of sphincter contraction or detrusor inhibition and thus an unreliable pressure reading in the condom. For a successful condom pressure measurement, it is necessary that the condom is quickly filled and pressurized. The time necessary to reach the maximum condom pressure mainly depends on the flow rate. We re-analyzed the data and found that a free flow rate above 5.4 ml/s may be considered a requirement for the condom pressure to accurately reflect the bladder pressure.[10] That does not imply that condom pressure measurements are impossible at lower flow rates, it just means that at flow rates below 5.4 ml/s in our data more than half of the measurements did not yield a reliable pressure value. Of course, it may be wondered if pressure measurements are diagnostically necessary at such low flow rates.

## APPLICATION OF THE CONDOM CATHETER METHOD IN AN EPIDEMIOLOGICAL STUDY

Apart from its application in the clinical setting, for instance for diagnosing and grading infravesical obstruction, the condom catheter method also enables epidemiological urodynamic studies in volunteers which are not, or hardly, possible using invasive urodynamic methods. We are currently doing such a study on changes in urethral resistance and urinary bladder contractility as a function of age related prostatic enlargement. As the study also leads to a number of conclusions relating to the performance of the non-invasive method, we will shortly discuss some of its results here.

### Recruitment of non-invasive epidemiological study

As benign prostatic enlargement (BPE) develops over a very long period of time, studying its effects on the urinary bladder makes it necessary to regularly evaluate a group of (otherwise) healthy males during 40 years (ages 40-80). We have chosen a more practical implementation by following a number of age-stratified cohorts during a shorter period. 8 cohorts, with initial ages of 38-42, 43-47, 48-52, 53-57, 58-62, 63-67, 68-72, and 73-77 have been recruited. Each cohort is followed for five years, and is non-invasively studied three times: at recruitment, after 2.5 years, and after 5 years. Presently, two of these evaluations have been completed, and the third is being done. In total 1020 male healthy volunteers have been recruited, mainly by general practitioners (GPs) in the community of Schiedam, near Rotterdam, The Netherlands, between November 2001 and December 2003. The Medical Ethical Committee of Erasmus MC approved the study. Exclusion criteria were: unable to urinate in a standing position, previous lower urinary tract surgery, congenital disease of the lower urinary tract, use of medication or other interventions for LUTS, other diseases that could alter urinary function (e.g., Parkinson, CVA, DM, kidney failure, bladder/prostate cancer, current urinary tract infection) and heart failure (the volunteers had to drink a lot of water to fill the bladder between voidings). Later, volunteers using anticoagulants were also excluded as we found that in 7% of the volunteers slight, self-terminating haematuria occurred after/during the non-invasive measurements. Factors influencing the recruitment of the volunteers were analyzed,[11] and the recruited population was compared to another proven representative population. It was concluded that the recruited population was not urologically different from the general population.

### Results of epidemiological study: Reproducibility, Reliability, Resistance and Residual volume

#### Reproducibility

In 95% of the volunteers, at least one successful condom pressure measurement could be done in the first evaluation round. In 967 volunteers (90%) two (or more) condom pressure measurements were done. The reproducibility of the method was analyzed using the method of Bland and Altman.[12] The standard deviation of the difference between the two pressure measurements was 18 cmH_2_O.[13] For lack of a standard to decide if this reproducibility is good or bad, we developed a method for comparing the reproducibility of different methods for clinical measurement.[14] We normalized the standard deviation of differences between two measurements by dividing it by the difference of the 97.5 and the 2.5 percentile of the mean of those two measurements, resulting in a relative standard deviation of 0.15. This reproducibility was comparable to, or slightly better than, that of pressure-flow parameters derived from a comparable population of patients studied with conventional invasive urodynamic methods.

### Reliability: The volume dependence of condom pressure measurements

As illustrated in Figure 2, voiding was usually interrupted several times during the non-invasive measurement. In a small group of volunteers it was established that the interruption did not influence the remainder of the voiding.[6] In the present study, the (bladder) volume dependence of the measured condom pressure was studied.[15] It was found that there is an optimum bladder volume for isovolumetric pressure measurements, averaging 264±122 ml (mean ± standard deviation). Measurements should be taken at or above the optimum volume. At volumes below the optimum volume, the pressure decreases by approximately 5% for each 10% of volume decrease. At bladder volumes smaller than 247 ml pressure readings in 50% of subjects are suboptimal. The optimum volume for isovolumetric pressure generation was only marginally related to voiding diary parameters. Probably it represents mechanical properties of the bladder, whereas voiding diary parameters more likely represent neurophysiologic properties, such as sensory thresholds. However, the optimum volume does not reflect the optimum (smooth) muscle length for force generation of the bladder wall: during normal voiding bladder smooth muscle always operates at a suboptimal length for force generation.

### Resistance of the urethra

In a separate analysis, an approximation of the urethral resistance was estimated from the maximum condom pressure and the maximum free flow rate (measured separately) in 667 of the volunteers.[16] 28% (185/667) of these had a high non-invasively estimated urethral resistance, and these volunteers had a significantly higher International Prostate Symptom Score (IPSS; mean ± SD) of 7.3 ± 5.2 than those with a low urethral resistance (IPSS: 5.7 ± 4.6), Mann-Whitney U-test: *P* < 0.001. IPSS and urethral resistance were significantly correlated, Spearman's rho 0.20, *P* < 0.001. The prostate volumes, 36 ± 21 cm3 in the high resistance versus 33 ± 17 cm3 in the low resistance group, did not differ significantly, P = 0.18. It was concluded that an elevated urethral resistance is a necessary, but not a sufficient condition for LUTS.

#### Residual Volume

In the second evaluation round, post void residual (PVR) volume was measured using transabdominal ultrasound immediately after each voiding to study the influence of the condom catheter measurements on bladder emptying. Volunteers had to void once in a flow meter to determine the maximum urinary flow rate and then underwent two condom catheter measurements for measurement of the isovolumetric bladder pressure.

In 149 volunteers, PVR volume [median (inter-quartile range)] after free voiding with a voided volume of 375 ± 192 ml (mean ± SD), was 36 ml (8-74 ml). After the two condom measurements PVR volume was significantly increased to 63 ml (37-120) and 83 ml (43-147), respectively (Wilcoxon Rank test, both *P*< 0.01). There are several possible explanations for this doubling in PVR volume. To test these hypotheses groups of subsequent volunteers in the second evaluation round were subjected to slightly modified protocols.

According to the standard protocol, voiding was interrupted as many times as feasible during the bladder pressure measurements. We hypothesized that these involuntary interruptions could influence the performance of the bladder and result in incomplete emptying of the bladder. When the measurements were stratified into those with 3 or less interruptions, and those with more than 3, no difference in PVR volume after the first condom catheter measurement was found 52 (30-108) ml vs. 71 (42-133) ml, respectively (Mann Whitney U test, P=0.191). After the second condom catheter measurement, 4 or more interruptions resulted in a significantly larger PVR 70 (40-138) ml vs. 105 (57-167) ml, respectively (Mann Whitney U test, P=0.048). However, when more interruptions are made this is usually because the voided volume is larger so that the voiding takes longer.

When the voided volume was taken into account, analysis of variance showed no significant influence of the number of interruptions of voiding. Thus, the number of interruptions had no influence on PVR volume.

A second possible reason for increased PVR volume after condom catheter measurements is that the volunteers had to void three times in a very short period (2-3 h). The rapid filling of the bladder and the increased voiding frequency could exhaust the bladder muscle and result in less effective emptying at the end of the protocol. Therefore, the subsequent 103 volunteers were asked to start with the two condom measurements and do the free voiding at the end of the protocol. PVR volume after free voiding was not significantly different when free voiding was done as a first or last test 36 (8-74) ml vs. 30 (6-71) ml respectively (Mann Whitney U test, P=0.778), suggesting that the bladder was not exhausted during the protocol and could still empty effectively at the end of the session.

Thirdly, to decrease the waiting time during the examination, diuresis was forced by drinking almost 1½ liter fluid within a few hours. Although forced diuresis does not alter uroflowmetric parameters like maximal flow rate and voided volume,[17] in one study PVR volume was found to be larger after increased water load diuresis.[18] Therefore, in the above-described group of 103 volunteers who did the free voiding at the end of the measurement session, the fluid intake before the first voiding was noted. If a volunteer had not been drinking extra fluid to increase diuresis before the first voiding, this was noted as “normal fluid intake”. If extra fluid was taken at home to increase diuresis or when the volunteer started drinking the extra fluid in the outpatient clinic before the first voiding, it was noted as “increased fluid intake”. When diuresis was forced by increased fluid intake (n=82) the PVR volume after the condom catheter measurement was significantly larger than when diuresis was normal (n=20); 56 (24-126) ml vs. 21 (2-76) ml respectively (Mann Whitney U test, *P*=0.032).

## CONCLUSION

We conclude that bladder pressure can successfully be measured non-invasively using the condom catheter method. The reproducibility of the measured isovolumetric bladder pressure is comparable to that of conventional pressure-flow parameters. The measured pressure can be used to diagnose bladder outlet obstruction with a diagnostic accuracy of 0.98, which compares most favorably with the accuracy of 0.79 for Q_max_ in the same population. During condom catheter measurements, both the involuntary interruption of voiding and the forced diuresis increase post void residual (PVR) volume. However, the ineffective emptying is not caused by bladder exhaustion during the examination and seems independent of the number of interruptions. The increase in PVR volume does not affect the accuracy of the pressure measurements, but PVR volume measured after condom catheter measurements should not be used as a diagnostic parameter.
